# Association of Physical Activity with Psychological Distress and Poor Mental Health Days Among Informal Caregivers of Older Adults: Findings from the 2023 BRFSS Caregiver Module

**DOI:** 10.3390/healthcare14172734

**Published:** 2026-08-27

**Authors:** Damián Pereira-Payo, Raquel Pastor-Cisneros, María Mendoza-Muñoz, Amparo Rodríguez-Gutiérrez

**Affiliations:** 1 Health, Economy, Motricity and Education (HEME) Research Group, Faculty of Sport Sciences, University of Extremadura, 10003 Caceres, Spain; 2 Physical Activity for Education, Performance and Health (PAEPH) Research Group, Faculty of Sport Sciences, University of Extremadura, 10003 Caceres, Spain; 3 Department of Communication and Education, Universidad Loyola Andalucía, 41014 Sevilla, Spain; 4 Research Group on Didactic and Behavioural Analysis in Sport, Faculty of Sports Sciences, University of Extremadura, 10003 Caceres, Spain

**Keywords:** psychological distress, mental health, caregiving, informal caregivers, physical inactivity, WHO physical activity guidelines

## Abstract

**Highlights:**

**What are the main findings?**

- Physical inactivity was significantly associated with poorer self-reported mental health indicators among informal caregivers of older adults in a survey-weighted BRFSS caregiver-module sample from participating US jurisdictions.
- Inactive caregivers showed higher odds of self-reported mental health indicators than those meeting both aerobic and muscle-strengthening physical activity recommendations.

**What are the implications of the main findings?**

- Complete physical inactivity may be a marker of poorer self-reported mental health among informal caregivers of older adults.
- Caregiver support programs could consider low-burden, flexible physical activity options, while longitudinal and intervention studies are needed to determine whether increasing activity improves mental health in this population.

**Abstract:**

**Background/Objectives:** Informal caregivers of older adults are a high-burden population for psychological problems, yet evidence on the relationship between physical activity (PA) and mental health in this population is still limited. The aim of this work is to examine the association between PA status and self-reported mental health indicators (psychological distress and the number of poor mental health days) among caregivers of older adults. **Methods:** This cross-sectional study used data from the 2023 Behavioral Risk Factor Surveillance System (BRFSS) caregiver module. The analytical sample included 1785 informal caregivers of older adults whose care recipients’ main condition was old age/infirmity/frailty or Alzheimer’s disease, dementia, or another cognitive impairment disorder. Physical activity was classified into three groups: meeting both aerobic and muscle-strengthening recommendations, reporting some PA but not meeting both recommendations, and being inactive. The self-reported mental health indicators were psychological distress (≥14 poor mental health days in the past 30 days) and the number of poor mental health days. Survey-weighted logistic regression and quasi-Poisson regression models were fitted, adjusting for sociodemographic, health-related, and caregiving-related covariates. **Results:** The weighted prevalence of psychological distress was 18.8%, and the mean of poor mental health days was 5.99 days in the previous 30 days. The weighted prevalence of psychological distress was 16.3% among caregivers meeting both recommendations, 18.2% among those reporting some PA, and 23.2% among inactive caregivers; however, differences across PA groups were not statistically significant. The weighted mean number of poor mental health days was 5.5, 6.2, and 6.1 days, respectively, with no significant differences across PA groups. In adjusted logistic regression models, physically inactive caregivers had higher odds of psychological distress than those meeting both activity recommendations (OR = 3.10, 95% CI: 1.58–6.09; *p* = 0.001). In adjusted quasi-Poisson models, physically inactive caregivers also reported more poor mental health days (RR = 1.51, 95% CI: 1.08–2.10; *p* = 0.016) compared to those who met both recommendations. No significant differences were observed for caregivers who met only aerobic recommendations or only muscle-strengthening recommendations, or had insufficient physical activity relative to the reference group. **Conclusions:** In this survey-weighted BRFSS caregiver-module sample from participating US jurisdictions, physical inactivity was associated with poorer self-reported mental health indicators among caregivers of older adults. These findings suggest that physical inactivity may help identify caregivers at higher risk of poorer self-reported mental health, although longitudinal and intervention studies are needed to clarify directionality and causality.

## 1. Introduction

The aging population in the United States has resulted in an increase in the number of older adults requiring assistance with daily living, thereby leading to a growing reliance on informal caregivers [1]. The provision of unpaid care by family members and friends is of paramount importance in ensuring the physical, emotional and social well-being of older adults [2]. Nevertheless, caregiving responsibilities are frequently associated with considerable psychological, emotional and physical strain [3]. As demonstrated in preceding studies, caregivers frequently exhibit elevated levels of stress, anxiety, depression and general psychological distress in comparison to non-caregivers [4]. These challenges may be exacerbated by the duration and intensity of caregiving duties, financial strain, social isolation, and limited opportunities for self-care [5].

Consequently, the mental health of caregivers has emerged as a significant public health concern. The impact of psychological distress on caregivers’ quality of life is a multifaceted issue, with the potential to compromise both their well-being and their capacity to deliver effective and sustainable care [6]. It is imperative to ascertain the protective factors that can mitigate distress and enhance mental well-being among caregivers, in order to formulate interventions and public health strategies aimed at supporting this vulnerable population [7].

There is a broad consensus that physical activity is a significant predictor of mental health. Regular engagement in physical activity has been shown to be associated with a reduction in symptoms of depression and anxiety, an improvement in mood, a decrease in stress levels, and an enhancement of psychological resilience [8]. The mechanisms underlying these benefits include physiological effects such as improved cardiovascular health and neurochemical regulation, as well as psychosocial benefits including social interaction, increased self-efficacy, and stress reduction [9]. Notwithstanding the existence of this evidence, caregivers frequently encounter obstacles that impede their ability to engage in regular physical activity, which may be attributed to factors such as limited time, fatigue, and competing caregiving responsibilities [10,11].

Beyond the well-established benefits of physical activity, accumulating evidence suggests that physical inactivity may be one of the strongest behavioral markers associated with poor mental health outcomes. Population-based studies have consistently shown that physically inactive individuals report higher levels of psychological distress, depression, anxiety, and poorer mental health than those engaging in any amount of physical activity, with inactivity representing the profile associated with the greatest mental health burden [12,13]. Moreover, a dose–response relationship has been reported, whereby increasing levels of physical activity, from low to moderate and high, are associated with progressively better mental health outcomes, suggesting that even modest amounts of physical activity may confer psychological benefits compared with complete inactivity [14]. Similar associations have also been observed across specific population groups, including smokers, ex-smokers, and individuals with depression, reinforcing the role of physical inactivity as a potential modifiable risk factor for poor mental health [15,16]. Although evidence in caregivers remains limited, recent findings indicate that physically inactive caregivers are also more likely to experience poorer mental health outcomes than their physically active counterparts [17]. Therefore, identifying physically inactive caregivers may be particularly relevant, as this subgroup could represent those at the greatest risk of psychological distress and related mental health problems. Despite the fact that a number of studies have examined the relationship between physical activity and mental health in the general population, fewer investigations have specifically focused on caregivers of older adults using large population-based survey data [2,18]. It is imperative to comprehend this association within caregiving populations, as caregivers may encounter distinctive stressors and health behaviors that influence both physical activity participation and mental health.

Given the growing number of informal caregivers and the substantial mental health burden associated with caregiving, identifying lifestyle-related markers associated with poorer self-reported mental health is of considerable public health importance. Physical activity has been consistently associated with better mental health in the general population; however, evidence regarding its relationship with psychological distress among caregivers of older adults remains limited. Therefore, the aim of the present study was to examine the association between physical activity status and self-reported mental health indicators among caregivers of older adults using data from the Behavioral Risk Factor Surveillance System (BRFSS). Specifically, we investigated whether caregivers who met both aerobic and muscle-strengthening recommendations, those who reported some physical activity but did not meet both recommendations, and those who were physically inactive differed in psychological distress and the number of poor mental health days. We hypothesized that physically inactive caregivers would show higher odds of psychological distress and a greater number of poor mental health days than those meeting both aerobic and muscle-strengthening recommendations. Exploratory analyses using a more detailed five-category physical activity classification were conducted to examine whether this association remained consistent when partial compliance with aerobic or muscle-strengthening recommendations and insufficient physical activity were considered separately.

## 2. Materials and Methods

### 2.1. Study Design

This study employed a cross-sectional design using data from the 2023 Behavioral Risk Factor Surveillance System (BRFSS), a nationwide health-related telephone survey conducted annually by the Centers for Disease Control and Prevention (CDC) in collaboration with U.S. state and territorial health departments. The BRFSS is designed to collect information on health behaviors, chronic conditions, and preventive health practices among non-institutionalized adults aged 18 years and older in the United States.

The BRFSS questionnaire includes a core component administered across jurisdictions, optional modules selected by individual jurisdictions, and state-added questions. The caregiver module used in the present study was an optional module and was therefore not administered to all respondents in the 2023 BRFSS dataset. According to CDC documentation, in 2023 the caregiver module was administered in Arizona, Arkansas, Hawaii, Idaho, Louisiana, Oregon, Tennessee, and Texas in the combined landline and cell phone dataset, and in specific questionnaire versions for Maine, Iowa, Maryland, Ohio, and New York.

Accordingly, the source population for the present analysis consisted of non-institutionalized adults from BRFSS jurisdictions and questionnaire versions that administered the 2023 caregiver module. For this reason, the weighted estimates should be interpreted as applying to adults represented by these participating caregiver-module jurisdictions and questionnaire versions, rather than as unqualified nationally representative estimates for all informal caregivers in the United States.

### 2.2. Participants

The inclusion criteria for the study were:

- Being an informal caregiver (responding “Yes” to item CAREGIV1) for a person whose primary health problem is “Old age/infirmity/frailty” or “Alzheimer’s disease, dementia, or another cognitive impairment disorder” (item CRGVPRB3).
- Having complete data available for the items: MENTHLTH, _PAREC3, _TOTINDA, _AGE80, SEXVAR, _RACE, EDUCA, INCOME3, MARITAL, _BMI5, SMOKE100 and _RFDRHV8.

The complete 2023 BRFSS sample consisted of 433,323 participants. Of these, 421,821 were excluded because they were not informal caregivers, while 8707 were excluded because the primary health condition of the individuals they cared for was neither “Old age/infirmity/frailty” nor “Alzheimer’s disease, dementia, or another cognitive impairment disorder.” Finally, 856 participants were excluded due to missing information in the above-mentioned items.

**Figure 1:**
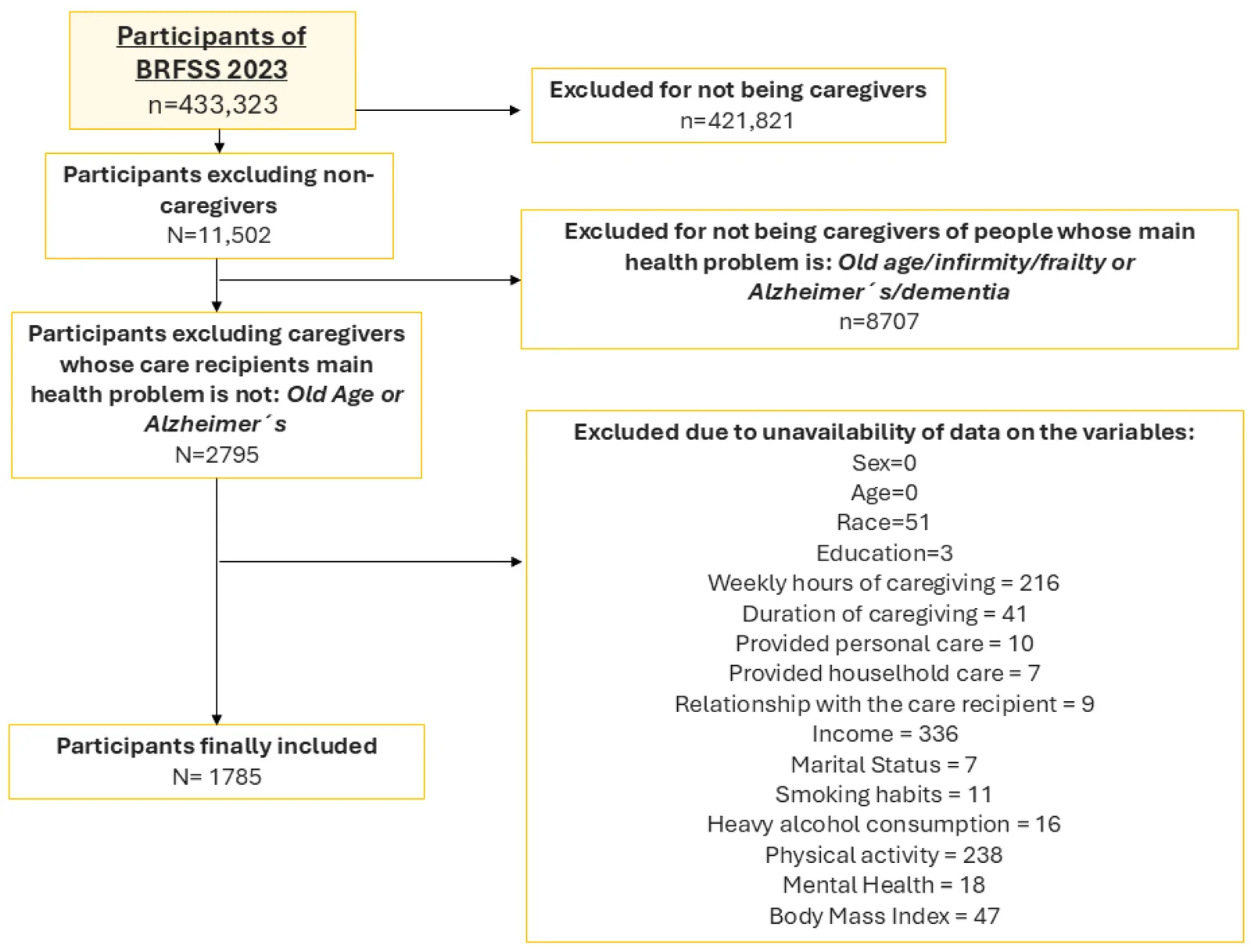
Flow diagram of the selection of informal caregivers of older adults from the 2023 BRFSS caregiver module.

The final analytical sample comprised 1939 participants (n = 1096 females and n = 843 males) (Figure 1). In addition, Supplementary Table S1 reports the missingness pattern for each analytical variable, and Supplementary Table S2 compares the characteristics of included and excluded otherwise eligible caregivers.

### 2.3. Variables

#### 2.3.1. Psychological Distress

Psychological distress was assessed using the BRFSS item MENTHLTH, which asks respondents *“Now thinking about your mental health, which includes stress, depression, and problems with emotions, for how many days during the past 30 days was your mental health not good?”*. Responses ranged from 0 to 30 days.

A dichotomous variable of psychological distress was created using the commonly applied BRFSS threshold of ≥14 days of poor mental health in the past 30 days, indicating frequent mental distress [19]. Participants reporting fewer than 14 days were classified as not having psychological distress.

In addition to the dichotomous outcome, the number of poor mental health days (0–30 days) was analyzed as a continuous count outcome in quasi-Poisson regression models.

These variables should therefore be interpreted as binary and count-based representations of the same self-reported mental health item, rather than as distinct mental health constructs.

#### 2.3.2. Physical Activity

Physical activity was assessed using BRFSS calculated variables derived from the physical activity module. These variables are based on leisure-time physical activity or exercise performed during the past 30 days, excluding activity performed as part of regular work. Two BRFSS calculated variables were used to define the physical activity status. The variable _PAREC3 classifies participants according to whether they meet aerobic and muscle-strengthening World Health Organization (WHO) physical activity recommendations. Aerobic recommendations were defined as participation in at least 150 min per week of moderate-intensity physical activity, or an equivalent amount of vigorous-intensity activity, whereas muscle-strengthening recommendations were defined as participation in muscle-strengthening activities on two or more days per week [20]. The variable _TOTINDA identifies participants reporting no leisure-time physical activity or exercise in the past 30 days.

For the main analyses, physical activity was classified into three mutually exclusive groups: (a) met both, (b) some physical activity, and (c) inactive. Participants were classified as “met both” if they met both aerobic and muscle-strengthening recommendations according to _PAREC3. The “some physical activity” group included participants who reported leisure-time physical activity but did not meet both recommendations, including those who met only the aerobic recommendation, met only the muscle-strengthening recommendation, or did not meet either recommendation but were not classified as inactive. Participants were classified as “inactive” if they were identified in _TOTINDA as reporting no physical activity or exercise in the past 30 days.

This three-category classification was selected as the primary exposure to distinguish complete physical inactivity from any non-zero leisure-time physical activity while preserving a guideline-based reference group. Participants who met both recommendations served as the reference group in the regression models because they represented the profile most consistent with current physical activity guidelines.

Exploratory analyses were also conducted using a more detailed five-category physical activity classification: met both recommendations, met aerobic recommendations only, met muscle-strengthening recommendations only, insufficient physical activity, and inactive. In this classification, the insufficient physical activity group included participants classified in _PAREC3 as not meeting either recommendation but who were not classified as inactive in _TOTINDA. These exploratory analyses were used to examine whether the main findings were consistent when partial compliance with aerobic or muscle-strengthening recommendations and insufficient but non-zero physical activity were considered separately.

#### 2.3.3. Caregiving

Caregiving-related characteristics were obtained from the BRFSS caregiver module and included as covariates in the adjusted models. “Weekly hours of caregiving” (CRGVHRS1) were used as an indicator of caregiving intensity, and categorized as: up to 8 h per week, 9 to 19 h per week, 20 to 39 h per week, and 40 h or more. “Duration of caregiving” (CRGVLNG1) reflected the length of time the respondent had been providing care and was categorized as: less than 30 days, 1 month to less than 6 months, 6 months to less than 2 years, 2 years to less than 5 years, and 5 or more years. The type of care provided was assessed using two dichotomous variables indicating whether the respondent provided personal care (CRGVPER1), such as assistance with feeding, bathing, dressing, or medication management, and whether the respondent provided household care (CRGVHOU1), such as managing finances, preparing meals, or cleaning. Relationship to the care recipient was derived from CRGVREL4 and categorized as parent, spouse or partner, other relative, and non-relative or family friend. The “other relative” category included parents-in-law, children, siblings or in-laws, grandparents, grandchildren, and other relatives. Responses of “don’t know/not sure” and “refused” were treated as missing.

#### 2.3.4. Age

Age was recorded in years and included as a continuous variable using the BRFSS variable _AGE80, which represents respondents’ age at the time of the survey.

#### 2.3.5. Sex

Sex was categorized as male or female according to the BRFSS variable SEXVAR.

#### 2.3.6. Race/Ethnicity

Race and ethnicity were classified using the BRFSS calculated variable _RACE. Participants were categorized as White non-Hispanic, Black non-Hispanic, American Indian or Alaska Native, Asian, Native Hawaiian or Pacific Islander, Other race, Multiracial, or Hispanic. White non-Hispanic participants were used as the reference group in the analysis.

#### 2.3.7. Educational Level

Educational attainment was determined using the BRFSS calculated variable EDUCA and categorized into five levels: elementary education, some high school, high school graduate, some college or technical school, and college graduate. The category “Never attended school or only kindergarten,” included in the original EDUCA variable, was excluded because it contained no participants (n = 0).

#### 2.3.8. Household Income

Annual household income was obtained from the BRFSS variable INCOME3 and categorized into eleven income groups ranging from <$10,000 to ≥$200,000. The $50,000–<$75,000 category was used as the reference group in regression models.

#### 2.3.9. Marital Status

Marital status was assessed using the BRFSS variable MARITAL and categorized as married, divorced, widowed, separated, never married, or a member of an unmarried couple. Married participants were used as the reference group in the analyses.

#### 2.3.10. Body Mass Index

Body mass index (BMI) was derived from self-reported height and weight in the BRFSS and calculated as weight in kilograms divided by height in meters squared (kg/m^2^) (item _BMI5). BMI was included as a continuous covariate in regression models.

#### 2.3.11. Smoking Status

Smoking status was assessed using the BRFSS item SMOKE100, which asks participants whether they had smoked at least 100 cigarettes during their lifetime. Participants were classified as non-smokers or smokers, with non-smokers serving as the reference group in the regression analyses.

#### 2.3.12. Heavy Alcohol Consumption

Heavy alcohol consumption was assessed using the BRFSS derived variable _RFDRHV8, which classifies respondents according to the Centers for Disease Control and Prevention (CDC) definition of excessive alcohol consumption (more than 14 alcoholic drinks per week for men and more than 7 drinks per week for women). Participants were categorized as having or not having heavy alcohol consumption, with the latter serving as the reference group in the analyses.

### 2.4. Data Analysis

All analyses accounted for the complex sampling design of the Behavioral Risk Factor Surveillance System (BRFSS) by incorporating sampling weights (item _LLCPWT), primary sampling units (item _PSU), and stratification variables (item _STSTR), following the analytic guidelines of the Centers for Disease Control and Prevention. Survey-weighted procedures were used to obtain population-level estimates for the participating caregiver-module jurisdictions and questionnaire versions.

Participants with missing information in any outcome, exposure, sociodemographic covariate, health-related covariate, or caregiving-related covariate were excluded using complete-case analysis. The final analytical sample therefore included 1795 caregivers with complete information on all variables used in the main models. To assess the potential impact of missing data, the missingness pattern for each analytical variable is reported in Supplementary Table S1, and the characteristics of included and excluded otherwise eligible caregivers are compared in Supplementary Table S2.

Descriptive statistics were calculated using survey-weighted methods. Continuous variables are presented as weighted means with standard deviations (SDs), and categorical variables as unweighted counts with weighted percentages. Differences in the prevalence of psychological distress across PA groups were evaluated using the Rao–Scott adjusted chi-square test. Differences in the weighted mean number of poor mental health days across PA groups were evaluated using a survey-weighted Wald test derived from a quasi-Poisson regression model accounting for the complex BRFSS sampling design.

The main exposure was a three-category PA variable: meeting both aerobic and muscle-strengthening recommendations, reporting some PA but not meeting both recommendations, and being inactive. Participants meeting both recommendations served as the reference group in all regression models.

Survey-weighted binary logistic regression models were used to evaluate the association between PA group and psychological distress. Both crude and adjusted models were fitted. Crude models included the PA group as the only independent variable and did not include covariates. Adjusted odds ratios (ORs) with 95% confidence intervals were estimated.

To examine differences in the number of poor mental health days across PA groups, survey-weighted quasi-Poisson regression models with a log link were fitted. This approach was chosen to account for overdispersion in count data. Both crude and adjusted models were fitted. Crude models included the PA group as the only independent variable and did not include covariates. Adjusted rate ratios (RRs) and 95% confidence intervals (CIs) were estimated. Rate ratios represent the relative change in the expected number of poor mental health days associated with each predictor.

All multivariable models were adjusted for age (continuous), sex, race/ethnicity, educational attainment, household income, marital status, body mass index (continuous), smoking status, heavy alcohol consumption, weekly hours of caregiving, duration of caregiving, provided personal care, provided household care, and relationship to the care recipient.

Exploratory analyses were conducted using a more detailed five-category PA classification: meeting both recommendations, meeting aerobic recommendations only, meeting muscle-strengthening recommendations only, insufficient PA, and inactive. The same survey-weighted logistic and quasi-Poisson regression models were refitted using this five-category PA variable. These exploratory models are presented in Supplementary Tables S3 and S4, respectively.

All statistical analyses were performed using R software (version 4.3.3) with the survey package. Statistical significance was defined as a two-sided *p*-value < 0.05.

## 3. Results

Table 1 summarizes the characteristics of the study population (n = 1785). The weighted sample consisted predominantly of female caregivers (57.3%), with a mean age of 53.29 years (SD = 16.52). Most participants identified as White non-Hispanic (61.9%), followed by Hispanic (19.9%) and Black non-Hispanic (9.5%). Regarding education, the largest proportion reported some college or technical school education (36.9%), followed by college graduates (28.3%) and high school graduates (26.2%). Approximately 61.9% of participants were married. In terms of health behaviors, 60.4% were non-smokers and 90.8% reported no heavy alcohol consumption. With respect to PA, 30.8% met both aerobic and muscle-strengthening recommendations, 44.8% reported some physical activity, and 24.4% were classified as physically inactive. Overall, the weighted prevalence of psychological distress was 18.8% among caregivers. Participants reported a mean of 5.99 poor mental health days in the past month (SD = 9.81), and the mean body mass index was 29.04 kg/m^2^ (SD = 6.98).

**Figure 2:**
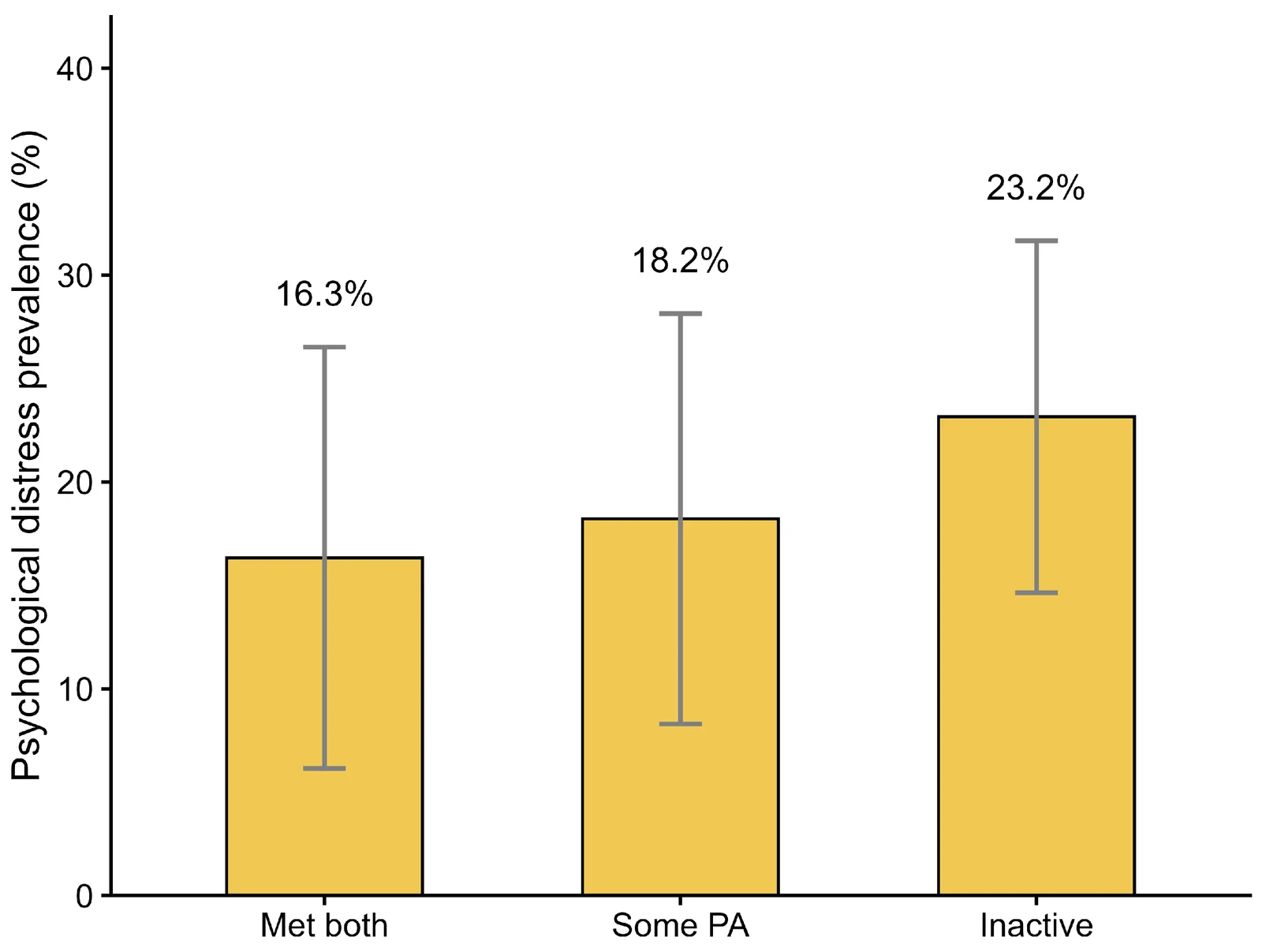
Weighted prevalence of psychological distress (≥14 poor mental health days in the past 30 days) across PA recommendation groups among caregivers of older adults.

Figure 2 shows the weighted prevalence of psychological distress across the three PA groups. Caregivers who met both aerobic and muscle-strengthening recommendations had a prevalence of psychological distress of 16.3%, while those reporting some PA had a prevalence of 18.2%. The highest prevalence was observed among physically inactive caregivers (23.2%). However, differences in psychological distress prevalence across PA groups were not statistically significant according to the Rao–Scott adjusted χ^2^ test (*p* = 0.676).

In survey-weighted crude logistic regression models, the physical activity group was not significantly associated with psychological distress. In the survey-weighted multivariable binary logistic regression model, physically inactive caregivers had significantly higher odds of psychological distress compared with those meeting both aerobic and muscle-strengthening PA recommendations (OR = 3.10; 95% CI: 1.58–6.09; *p* = 0.001). No significant association was observed for caregivers reporting some physical activity (OR = 1.50; 95% CI: 0.77–2.90; *p* = 0.234). Increasing age was associated with lower odds of psychological distress (OR = 0.96 per year; 95% CI: 0.94–0.98; *p* < 0.001), whereas female caregivers had higher odds of psychological distress compared with male caregivers (OR = 1.88; 95% CI: 1.08–3.28; *p* = 0.026). Hispanic caregivers had lower odds of psychological distress compared with White non-Hispanic caregivers (OR = 0.20; 95% CI: 0.08–0.49; *p* < 0.001). Household income was associated with psychological distress in selected income categories, particularly among caregivers earning $20,000–<$25,000 (OR = 4.44; 95% CI: 1.27–15.48; *p* = 0.019) and $25,000–<$35,000 (OR = 4.06; 95% CI: 1.49–11.08; *p* = 0.006), compared with the reference group ($50,000–<$75,000). Smoking (OR = 2.83; 95% CI: 1.63–4.91; *p* < 0.001) and heavy alcohol consumption (OR = 3.48; 95% CI: 1.30–9.34; *p* = 0.013) were also associated with higher odds of psychological distress. Among caregiving-related variables, providing 9 to 19 h of care per week was associated with higher odds of psychological distress (OR = 2.20; 95% CI: 1.15–4.20; *p* = 0.017), whereas caregiving for 2 years to less than 5 years (OR = 0.27; 95% CI: 0.11–0.67; *p* = 0.004) and being a non-relative or family friend caregiver (OR = 0.42; 95% CI: 0.19–0.93; *p* = 0.032) were associated with lower odds of psychological distress (Table 2).

In the survey-weighted crude logistic regression model, that is, the model including the physical activity group as the only independent variable and no covariates, the physical activity group was not significantly associated with psychological distress. Compared with caregivers meeting both aerobic and muscle-strengthening PA recommendations, those reporting some physical activity did not have significantly different odds of psychological distress (OR = 1.14; 95% CI: 0.43–3.02; *p* = 0.790), nor did physically inactive caregivers (OR = 1.54; 95% CI: 0.64–3.71; *p* = 0.333).

In exploratory analyses using a more detailed five-category PA classification, the association between physical inactivity and psychological distress remained consistent. Compared with caregivers meeting both aerobic and muscle-strengthening recommendations, physically inactive caregivers had significantly higher odds of psychological distress (OR = 3.12; 95% CI: 1.59–6.10; *p* < 0.001). In contrast, no significant associations were observed for caregivers meeting only aerobic recommendations (OR = 1.57; 95% CI: 0.79–3.11; *p* = 0.194), meeting only muscle-strengthening recommendations (OR = 1.81; 95% CI: 0.58–5.60; *p* = 0.305), or reporting insufficient PA (OR = 0.87; 95% CI: 0.20–3.66; *p* = 0.844) (Table S3). These exploratory findings support the main three-category analysis, suggesting that the association was primarily driven by complete physical inactivity rather than by intermediate PA profiles.

**Figure 3:**
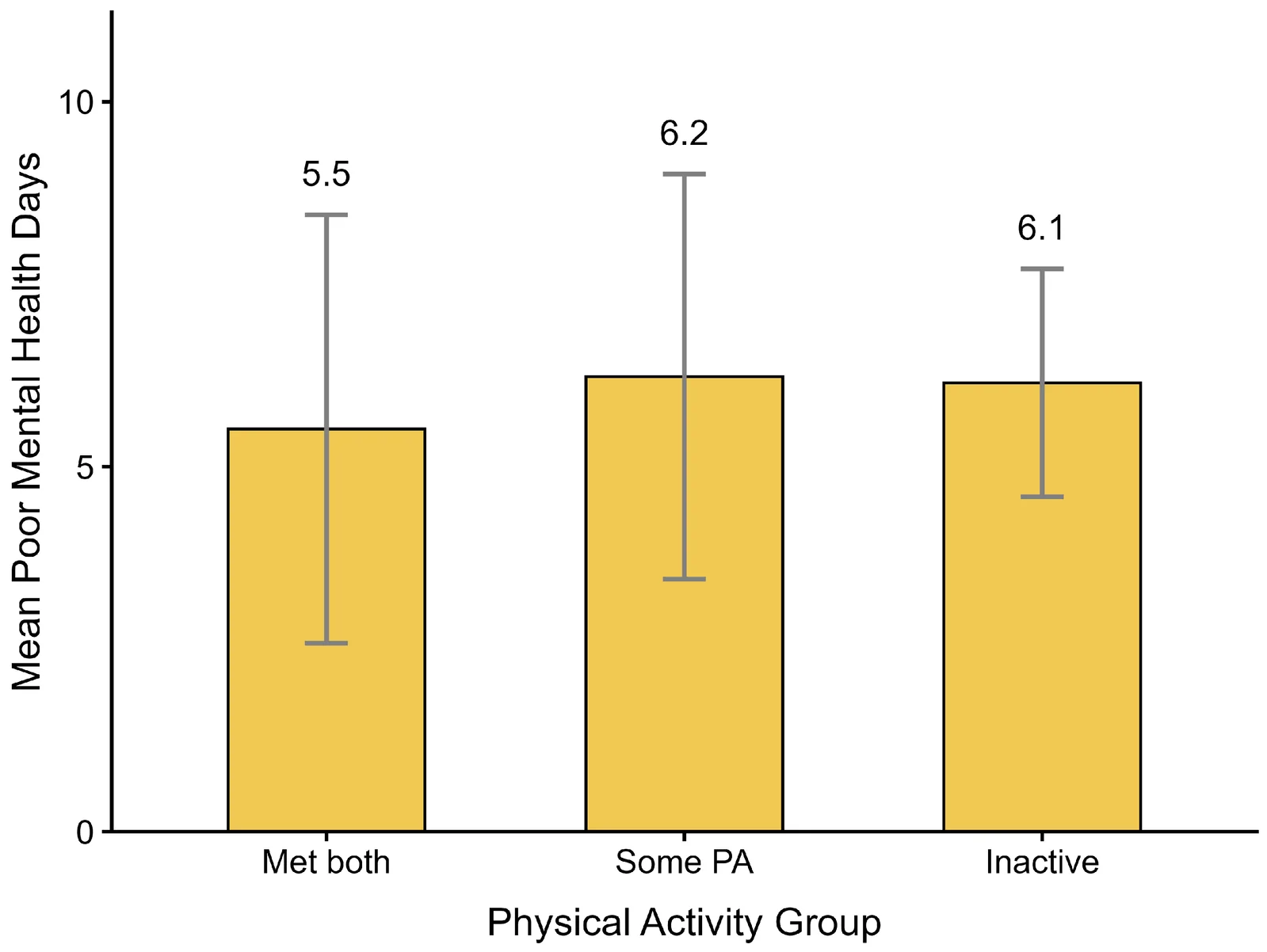
Weighted mean number of poor mental health days in the past 30 days according to PA level among caregivers of older adults. Error bars represent 95% confidence intervals calculated using the BRFSS complex survey design weights.

Figure 3 presents the weighted mean number of poor mental health days across the three PA groups. Caregivers meeting both aerobic and muscle-strengthening recommendations reported a mean of 5.5 poor mental health days, compared with 6.2 days among those reporting some physical activity and 6.1 days among physically inactive caregivers. Overall differences across PA groups were not statistically significant according to a survey-weighted Wald test derived from a quasi-Poisson regression model accounting for the complex BRFSS sampling design (F = 0.07; *p* = 0.928).

In survey-weighted quasi-Poisson regression models, physically inactive caregivers reported significantly more poor mental health days compared with those meeting both aerobic and muscle-strengthening PA recommendations (RR = 1.51; 95% CI: 1.08–2.10; *p* = 0.016), corresponding to approximately 51% more poor mental health days. No significant association was observed for caregivers reporting some physical activity (RR = 1.23; 95% CI: 0.89–1.70; *p* = 0.202). Age was inversely associated with poor mental health days (RR = 0.97 per year; 95% CI: 0.96–0.98; *p* < 0.001), indicating that older caregivers reported fewer days of poor mental health. Female caregivers reported significantly more poor mental health days than males (RR = 1.36; 95% CI: 1.06–1.76; *p* = 0.016). Hispanic caregivers reported fewer poor mental health days than White non-Hispanic caregivers (RR = 0.43; 95% CI: 0.27–0.67; *p* < 0.001). Higher educational attainment was associated with fewer poor mental health days in selected categories, particularly among caregivers with some high school education (RR = 0.36; 95% CI: 0.16–0.82; *p* = 0.016) and college graduates (RR = 0.40; 95% CI: 0.18–0.89; *p* = 0.024). Several household income categories were associated with more poor mental health days, including $15,000–<$20,000 (RR = 1.84; 95% CI: 1.17–2.89; *p* = 0.008), $20,000–<$25,000 (RR = 1.91; 95% CI: 1.13–3.24; *p* = 0.016), $25,000–<$35,000 (RR = 2.37; 95% CI: 1.55–3.62; *p* < 0.001), and $150,000–<$200,000 (RR = 1.81; 95% CI: 1.15–2.84; *p* = 0.010), compared with the reference group ($50,000–<$75,000). Smoking (RR = 1.69; 95% CI: 1.30–2.20; *p* < 0.001) and heavy alcohol consumption (RR = 1.55; 95% CI: 1.01–2.38; *p* = 0.045) were also associated with more poor mental health days. Among caregiving-related variables, caregiving for 2 years to less than 5 years (RR = 0.56; 95% CI: 0.37–0.85; *p* = 0.007) and being a caregiver of a non-relative or family friend (RR = 0.60; 95% CI: 0.40–0.90; *p* = 0.013) (Table 3) were associated with fewer poor mental health days.

In the survey-weighted crude quasi-Poisson regression model, which included the physical activity group without adjustment for covariates, the physical activity group was not significantly associated with the number of poor mental health days. Compared with caregivers meeting both aerobic and muscle-strengthening PA recommendations, those reporting some physical activity did not have significantly different rates of poor mental health days (RR = 1.13; 95% CI: 0.57–2.22; *p* = 0.723), nor did physically inactive caregivers (RR = 1.11; 95% CI: 0.62–2.00; *p* = 0.717).

Exploratory analyses using a more detailed five-category PA classification yielded consistent findings. Physical inactivity remained significantly associated with a higher number of poor mental health days compared with meeting both aerobic and muscle-strengthening recommendations (RR = 1.52; 95% CI: 1.09–2.11; *p* = 0.013). In contrast, no significant associations were observed for caregivers meeting only aerobic recommendations (RR = 1.31; 95% CI: 0.93–1.83; *p* = 0.119), meeting only muscle-strengthening recommendations (RR = 1.20; 95% CI: 0.74–1.96; *p* = 0.455), or reporting insufficient PA (RR = 0.92; 95% CI: 0.50–1.72; *p* = 0.803) (Table S4).

## 4. Discussion

This study examined the association between PA status and self-reported mental health indicators among informal caregivers of older adults using data from the 2023 BRFSS caregiver module, applying survey weights to account for the complex sampling design. The main analyses used a three-category PA variable that distinguished caregivers meeting both aerobic and muscle-strengthening recommendations, those reporting some PA but not meeting both recommendations, and those who were physically inactive. Descriptive survey-weighted comparisons showed that the prevalence of psychological distress and the mean number of poor mental health days did not differ significantly across PA groups. However, in adjusted regression models, physically inactive caregivers showed higher odds of psychological distress and a greater number of poor mental health days compared with those meeting both aerobic and muscle-strengthening recommendations. In contrast, caregivers reporting some PA did not differ significantly from the reference group in either outcome. Exploratory analyses using a more detailed five-category PA classification yielded consistent findings, suggesting that the observed associations were mainly driven by complete physical inactivity rather than by intermediate PA profiles. Beyond PA, poorer self-reported mental health indicators were also associated with several sociodemographic, health-related, and caregiving-related factors, including younger age, female sex, lower socioeconomic indicators, smoking, heavy alcohol consumption, caregiving hours, caregiving duration, and relationship to the care recipient. Overall, these findings partially support our hypothesis and suggest that complete physical inactivity may help identify caregivers with poorer self-reported mental health indicators, although the cross-sectional design prevents conclusions about directionality or causality.

The difference between the descriptive and adjusted results may be explained by the fact that they consider different information. Figure 2 and Figure 3 and the crude models compare PA groups without accounting for other participant characteristics, whereas the adjusted models also account for sociodemographic, health-related, and caregiving factors. In addition, the figures evaluate overall differences across all three PA groups, while the regression coefficients compare each group with caregivers meeting both recommendations. Previous research has identified several of these characteristics, including age, sex, socioeconomic circumstances, and caregiving burden, as relevant correlates of caregivers’ mental health [5,17,21,22,23]. If these characteristics were unevenly distributed across PA groups, their combined influence may have masked the association in the unadjusted analyses, making it more apparent after adjustment [24]. In the logistic regression model, adding covariates may also change the OR because crude and adjusted ORs are not directly equivalent [25]. However, because all covariates were entered simultaneously, the present analyses cannot determine which specific factor or combination of factors primarily accounted for this change.

These findings add to the growing evidence on the relationship between physical activity and mental health among carers. Previous research has shown that carers who are physically active tend to experience lower levels of depression, anxiety, stress and emotional overload, and a greater sense of psychological well-being than those who are physically inactive [26,27]. In line with this evidence, the present findings suggest that the most relevant differences in mental health may lie between completely inactive carers and those who engage in some level of regular physical activity. This pattern is consistent with recent research describing non-linear relationships between physical activity and mental health, indicating that the greatest differences are often observed when comparing complete inactivity with some level of physical activity, whereas further increases may yield smaller additional benefits [28,29].

Most of the available evidence on carers has compared physically active individuals with inactive ones or evaluated structured exercise programs such as walking, yoga, tai chi, and combined aerobic and strength training [26,30,31]. Taken together, these studies suggest that the most active carers experience lower levels of depression, anxiety, stress and emotional burden, as well as better quality of life and psychological well-being. However, systematic reviews also highlight considerable methodological heterogeneity and a lack of consensus regarding the optimal type or dose of physical activity for this group [32,33,34].

In this context, the results of the present study provide additional evidence by showing that the most consistent associations with poorer mental health were found specifically among physically inactive carers. In multivariable analyses, inactive carers had higher odds of psychological distress than those who met both aerobic and muscle-strengthening recommendations (OR = 3.10; 95% CI: 1.58–6.09), while caregivers reporting some physical activity but not meeting both recommendations did not differ significantly from the reference group. A similar pattern was observed for the number of poor mental health days, with inactive carers reporting a higher rate of poor mental health days than those meeting both recommendations (RR = 1.51; 95% CI: 1.08–2.10). These findings suggest that complete physical inactivity may be a relevant marker of poorer self-reported mental health among caregivers, although the cross-sectional design prevents determining whether inactivity contributes to poorer mental health, poorer mental health reduces physical activity, or both.

The observed findings may point not only to the potential relevance of physical activity, but also to the importance of considering prolonged inactivity and sedentary behavior in relation to caregivers’ mental health. Previous evidence suggests that sedentary time is independently associated with greater psychological distress, even when levels of physical activity are taken into account [35,36]. Some of these studies also indicate that carers have lower levels of physical activity and a higher risk of inactivity than non-carers, which could further increase their psychological vulnerability. A survey conducted in the United States found that carers who were sedentary for approximately six hours per day exhibited higher levels of mental stress, while among carers of cancer patients, prolonged periods of continuous inactivity (20–30 min) were associated with greater stress and poorer quality of life, regardless of the total time spent on physical activity [21,37]. Taken together, previous evidence suggests that sedentary behavior and prolonged inactivity are associated with poorer mental health among caregivers. However, the present study did not directly assess sedentary time; therefore, physical inactivity in our analyses should not be interpreted as a direct measure of sedentary behavior.

The very nature of caregiving may help to explain this pattern. Informal carers often report high levels of depression, anxiety, stress and emotional overload, as well as significant time constraints and fatigue, which can make it challenging to prioritize self-care behaviors such as regular physical activity [38,39,40]. Many carers describe feeling under constant pressure, which makes it hard for them to prioritize basic self-care activities such as resting, exercising, and attending to their own medical needs. From this perspective, the relationship between physical inactivity and mental health may be bidirectional: psychological distress and caregiver burden may reduce the capacity to maintain active lifestyles, while inactivity may also be linked to poorer psychological well-being through reduced opportunities for movement, social interaction, and stress regulation. Importantly, several studies have shown that flexible and home-based interventions, including walking programs, yoga and supervised moderate exercise, can improve mental health and caregiver burden even among previously sedentary carers, while observational evidence in Spanish informal caregivers indicates that any level of physical activity is associated with better self-perceived health, greater self-esteem, better coping and lower stress compared with inactivity [17,22,26]. Together, these findings support the need for longitudinal and intervention studies to determine whether feasible, caregiver-adapted strategies to address physical inactivity and incorporate regular movement into daily routines are followed by improvements in mental health, or whether poorer mental health primarily limits caregivers’ ability to remain active.

Finally, the results highlight the importance of social and socioeconomic factors in carers’ mental health. In the present study, female carers, smokers, individuals reporting heavy alcohol consumption, and caregivers in selected lower-income categories showed poorer self-reported mental health indicators. Multivariable analyses revealed that female sex was associated with higher odds of psychological distress and a greater number of poor mental health days, whereas older age was associated with better self-reported mental health indicators. Educational attainment showed associations with fewer poor mental health days in selected categories, although this pattern was not consistently observed for psychological distress. Furthermore, lower income levels, smoking and high alcohol consumption showed associations with poorer self-reported mental health indicators. Caregiving-related characteristics also appeared relevant: providing 9 to 19 h of care per week was associated with higher odds of psychological distress, whereas caregiving for 2 years to less than 5 years and being a non-relative or family friend caregiver were associated with fewer poor mental health indicators in the adjusted models. These findings are consistent with previous research showing greater psychological vulnerability among female caregivers, individuals with lower socioeconomic status, and younger caregivers, while also highlighting the important role of economic, educational, and gender inequalities in the mental health disparities observed in this population [23,41,42]. Taken together, these findings reinforce the idea that caregivers’ mental health is shaped not only by health-related behaviors such as physical activity, but also by broader social and contextual vulnerabilities. Although the revised models accounted for several caregiving-related characteristics, residual confounding by unmeasured aspects of caregiving burden, co-residence, care-recipient severity, and social support may remain. Therefore, the observed association between physical inactivity and poorer self-reported mental health should be interpreted as an association rather than evidence of a causal effect.

### 4.1. Strengths and Practical Applications

Firstly, the study used data from a large BRFSS caregiver-module sample of caregivers of older adults from participating US jurisdictions and questionnaire versions, which supports population-level inference within the scope of the administered module. Furthermore, the analyses accounted for the complex BRFSS survey design, including sampling weights, primary sampling units, and stratification variables, thereby providing more robust survey-weighted estimates. A further strength of the study lies in the detailed classification of physical activity profiles according to compliance with WHO recommendations, which allowed complete physical inactivity to be distinguished from insufficient but non-zero physical activity and partial compliance with aerobic or muscle-strengthening recommendations.

From a pragmatic standpoint, the findings suggest that physical inactivity may help identify caregivers with poorer self-reported mental health indicators. Therefore, caregiver support programs and future intervention studies may consider accessible forms of physical activity, such as walking, home-based exercise, or regular movement during daily caregiving routines, while formally evaluating their effects on caregivers’ mental health. The study also underscores the importance of paying particular attention to caregiver groups with poorer mental health profiles, including women and individuals with lower socioeconomic status.

### 4.2. Limitations and Future Line Research

Several limitations should be acknowledged. Firstly, the cross-sectional design prevents the establishment of causal relationships between physical activity and self-reported mental health indicators. Consequently, it remains uncertain whether inactivity contributes to poorer self-reported mental health, or whether poorer mental health reduces caregivers’ ability or motivation to engage in physical activity, or whether both are influenced by other unmeasured factors. Second, all variables were self-reported, which may have introduced recall bias and reporting inaccuracies. In addition, psychological distress and poor mental health days were both derived from the same BRFSS item (MENTHLTH). Therefore, they should not be interpreted as independent mental health constructs. Rather, they represent complementary binary and count-based operationalizations of the same self-reported mental health measure.

Although the adjusted models included several caregiving-related characteristics, including caregiving hours, caregiving duration, provided personal care, provided household care, and relationship to the care recipient, residual confounding remains possible. The available BRFSS variables did not fully capture some potentially relevant dimensions of caregiving, such as co-residence with the care recipient, objective severity of the care recipient’s functional or cognitive impairment, caregiver burden, or the availability of formal and informal support. These factors may influence both physical activity patterns and mental health indicators. Future research should use longitudinal and intervention-based designs to clarify directionality and evaluate whether changes in physical activity are followed by changes in caregivers’ mental health. Further studies should also examine the role of sedentary behavior and identify feasible physical activity strategies adapted to the needs and daily constraints of caregivers.

## 5. Conclusions

In this survey-weighted BRFSS caregiver-module sample from participating US jurisdictions, complete physical inactivity was associated with poorer self-reported mental health indicators among caregivers of older adults whose care recipients’ main condition was old age/infirmity/frailty or Alzheimer’s disease, dementia, or another cognitive impairment disorder. Caregivers reporting some physical activity did not differ significantly from those meeting both aerobic and muscle-strengthening recommendations. These findings suggest that physical inactivity may help identify caregivers at higher risk of poorer self-reported mental health, although longitudinal and intervention studies are needed to clarify directionality and causality.

## Figures and Tables

**Table 1:** Characteristics of the study population.

Variable		N	Weighted (%)
Sex	Male	773	42.7%
	Female	1012	57.3%
Race/Ethnicity	White non-Hispanic	1276	61.9%
	Black non-Hispanic	81	9.5%
	American Indian/Alaska Native	30	1.0%
	Asian	120	2.5%
	Native Hawaiian/Pacific Islander	27	0.3%
	Other race	14	1.9%
	Multiracial	91	3.1%
	Hispanic	146	19.9%
Education	Elementary education	15	2.5%
	Some high school	60	6.1%
	High school graduate	364	26.2%
	Some college/technical school	545	36.9%
	College graduate	801	28.3%
Household income	<$10,000	46	3.3%
	$10,000–<$15,000	36	2.1%
	$15,000–<$20,000	69	3.5%
	$20,000–<$25,000	88	5.5%
	$25,000–<$35,000	184	12.3%
	$35,000–<$50,000	275	14.3%
	$50,000–<$75,000	310	14.7%
	$75,000–<$100,000	267	11.6%
	$100,000–<$150,000	270	15.9%
	$150,000–<$200,000	123	9.3%
	$200,000 or more	117	7.3%
Marital status	Married	1093	61.9%
	Divorced	270	13.1%
	Widowed	129	3.7%
	Separated	29	2.6%
	Never married	214	14.5%
	Member of unmarried couple	50	4.3%
Smoking status	Smoker	755	39.6%
	Non-smoker	1030	60.4%
Heavy alcohol consumption	No	1665	90.8%
	Yes	120	9.2%
Physical activity group	Met both recommendations	568	30.8%
	Some physical activity	811	44.8%
	Inactive	406	24.4%
Psychological distress	No	1514	81.2%
	Yes	271	18.8%
Weekly hours of caregiving	Up to 8 h per week	943	48.1%
	9 to 19 h per week	274	19.6%
	20 to 39 h per week	220	13.0%
	40 h or more	348	19.3%
Duration of caregiving	Less than 30 days	183	13.8%
	1 month to less than 6 months	172	7.6%
	6 months to less than 2 years	418	19.4%
	2 years to less than 5 years	558	32.4%
	5 or more years	454	26.8%
Provided personal care	Yes	951	56.8%
	No	834	43.2%
Provided household care	Yes	1451	79.7%
	No	334	20.3%
Relationship to care recipient	Parent	821	48.5%
	Spouse/partner	272	9.3%
	Other relative	409	28.5%
	Non-relative/family friend	283	13.7%
**Variable**		**Mean**	**SD**
Age (years)		53.29	16.52
Body mass index		29.04	6.98
Poor mental health days		5.99	9.81

n represents unweighted sample counts. % are weighted to account for the complex sampling design of the BRFSS. SD = standard deviation.

**Table 2:** Survey-weighted binary logistic regression model for psychological distress (≥14 poor mental health days in the past 30 days) among caregivers of older adults.

Variable	Category	Odds Ratio	(95% CI)	*p*-Value
**Physical activity group** (ref: *Met both*)	Some physical activity	1.50	0.77–2.90	0.234
	Inactive	3.10	1.58–6.09	0.001 *
**Sex** (ref: *Male*)	Female	1.88	1.08–3.28	0.026 *
**Race/ethnicity** (ref: *White non-Hispanic*)	Black non-Hispanic	0.46	0.16–1.29	0.141
	American Indian/Alaska Native	0.25	0.04–1.42	0.118
	Asian	0.69	0.15–3.12	0.625
	Native Hawaiian/Pacific Islander	0.78	0.08–7.60	0.830
	Other race	0.06	0.00–1.11	0.059
	Multiracial	1.22	0.40–3.70	0.731
	Hispanic	0.20	0.08–0.49	<0.001 *
**Education** (ref: *Elementary education*)	Some high school	0.24	0.05–1.27	0.094
	High school graduate	0.31	0.07–1.46	0.139
	Some college/technical school	0.44	0.09–2.12	0.307
	College graduate	0.22	0.04–1.09	0.064
**Household income** (ref: *$50,000–<$75,000*)	< $10,000	0.53	0.12–2.39	0.407
	$10,000–<$15,000	0.53	0.14–2.10	0.370
	$15,000–<$20,000	2.73	0.83–8.94	0.098
	$20,000–<$25,000	4.44	1.27–15.48	0.019
	$25,000–<$35,000	4.06	1.49–11.08	0.006
	$35,000–<$50,000	1.04	0.36–3.03	0.940
	$75,000–<$100,000	0.51	0.15–1.72	0.276
	$100,000–<$150,000	1.10	0.40–3.01	0.856
	$150,000–<$200,000	2.71	0.87–8.46	0.086
	$200,000 or more	0.87	0.17–4.56	0.869
**Marital status** (ref: *Married*)	Divorced	1.85	0.86–4.01	0.116
	Widowed	0.86	0.32–2.34	0.766
	Separated	1.69	0.28–10.04	0.564
	Never married	1.31	0.53–3.27	0.559
	Member of unmarried couple	0.69	0.20–2.34	0.551
**Smoking** (ref: *Non-smoker*)	Smoker	2.83	1.63–4.91	<0.001 *
**Heavy alcohol** **consumption** (ref: *No*)	Yes	3.48	1.30–9.34	0.013 *
**Weekly hours of caregiving** (ref: *Up to 8 h per week*)	9 to 19 h per week	2.20	1.15–4.20	0.017 *
	20 to 39 h per week	1.21	0.46–3.21	0.696
	40 h or more	0.95	0.45–1.98	0.884
**Duration of caregiving** (ref: *Less than 30 days*)	1 month to less than 6 months	0.50	0.18–1.38	0.181
	6 months to less than 2 years	0.46	0.20–1.07	0.073
	2 years to less than 5 years	0.27	0.11–0.67	0.004 *
	5 or more years	1.21	0.52–2.81	0.650
**Relationship to care recipient** (ref: *Parent*)	Spouse/partner	1.58	0.63–3.98	0.328
	Other relative	0.75	0.39–1.43	0.383
	Non-relative/family friend	0.42	0.19–0.93	0.032 *
**Provided personal care** (ref: *No*)	Yes	1.35	0.73–2.48	0.336
**Provided household care** (ref: *No*)	Yes	0.56	0.25–1.23	0.146
**Age (years)**		0.96	0.94–0.98	<0.001 *
**Body mass index**		1.03	1.00–1.06	0.052

OR = odds ratio; CI = confidence interval; ref = reference; * = *p*-value < 0.05. Crude models included the physical activity group as the only independent variable. The reference category is indicated in parentheses for categorical variables.

**Table 3:** Association between PA and number of poor mental health days among caregivers of older adults: results from a survey-weighted quasi-Poisson regression model.

Variable		Rate Ratio	(95% CI)	*p*-Value
**Physical activity group** (ref: *Met both*)	Met Aerobic	1.23	0.89–1.70	0.202
	Met Strength	1.51	1.08–2.10	0.016 *
	Insufficient PA	1.36	1.06–1.76	0.016 *
	Inactive	0.62	0.38–1.03	0.063
**Sex** (ref: *Male*)	Female	0.45	0.18–1.10	0.080
**Race/Ethnicity** (ref: *White non-Hispanic*)	Black non-Hispanic	0.73	0.35–1.49	0.384
	American Indian/Alaska Native	0.63	0.19–2.13	0.460
	Asian	0.12	0.01–1.26	0.078
	Native Hawaiian/Pacific Islander	1.02	0.59–1.77	0.932
	Other race	0.43	0.27–0.67	<0.001 *
	Multiracial	1.23	0.89–1.70	0.202
	Hispanic	1.51	1.08–2.10	0.016 *
**Education** (ref: *Elementary education*)	Some high school	0.36	(0.16–0.81)	0.013 *
	High school graduate	0.40	(0.19–0.83)	0.014 *
	Some college	0.58	(0.29–1.15)	0.120
	College graduate	0.31	(0.14–0.66)	0.002 *
**Income** (ref: *$50,000–<$75,000*)	<$10,000	0.93	0.44–1.99	0.856
	$10,000–<$15,000	1.04	0.62–1.73	0.884
	$15,000–<$20,000	1.84	1.17–2.89	0.008 *
	$20,000–<$25,000	1.91	1.13–3.24	0.016 *
	$25,000–<$35,000	2.37	1.55–3.62	<0.001 *
	$35,000–<$50,000	1.34	0.83–2.17	0.229
	$75,000–<$100,000	0.83	0.48–1.43	0.495
	$100,000–<$150,000	1.09	0.71–1.67	0.695
	$150,000–<$200,000	1.81	1.15–2.84	0.010 *
	$200,000 or more	1.01	0.41–2.46	0.986
**Marital status** (ref: *Married*)	Divorced	1.39	0.97–1.99	0.072
	Widowed	1.05	0.61–1.80	0.858
	Separated	1.28	0.65–2.51	0.477
	Never married	1.25	0.83–1.89	0.288
	Unmarried couple	0.71	0.36–1.40	0.323
**Smoking** (ref: *Non-smoker*)	Smoker	1.69	1.30–2.20	<0.001 *
**Heavy alcohol consumption** (ref: *No*)	Yes	1.55	1.01–2.38	0.045 *
**Weekly hours of caregiving** (ref: *Up to 8 h per week*)	9 to 19 h per week	1.34	0.99–1.80	0.055
	20 to 39 h per week	1.10	0.71–1.71	0.663
	40 h or more	0.89	0.62–1.29	0.547
**Duration of caregiving** (ref: *Less than 30 days*)	1 month to less than 6 months	0.70	0.42–1.14	0.151
	6 months to less than 2 years	0.71	0.48–1.05	0.087
	2 years to less than 5 years	0.56	0.37–0.85	0.007 *
	5 or more years	1.16	0.78–1.72	0.469
**Relationship to care recipient** (ref: *Parent*)	Spouse/partner	1.20	0.76–1.87	0.433
	Other relative	0.86	0.65–1.15	0.316
	Non-relative/family friend	0.60	0.40–0.90	0.013 *
**Provided personal care** (ref: *No*)	Yes	1.20	0.89–1.60	0.231
**Provided household care** (ref: *No*)	Yes	0.79	0.55–1.13	0.198
**Age (years)**		0.97	0.96–0.98	<0.001 *
**Body mass index**		1.01	1.00–1.02	0.057

Ref = reference, CI = confidence interval; * = *p*-value < 0.05. Rate ratios (RRs) represent the relative change in the expected number of poor mental health days associated with each predictor variable. Crude models included the physical activity group as the only independent variable.

## Data Availability

The data presented in this study are openly available in the Centers for Disease Control and Prevention (CDC) Behavioral Risk Factor Surveillance System (BRFSS) at https://www.cdc.gov/brfss/annual_data/annual_2023.html (accessed on 11 February 2026).

## Supplementary Materials

The following supporting information can be downloaded at https://www.mdpi.com/article/10.3390/healthcare14172734/s1. Table S1: Missingness pattern among otherwise eligible caregivers (n = 2795); Table S2: Comparison of included complete-case participants and otherwise eligible caregivers excluded due to missing data; Table S3: Exploratory survey-weighted binary logistic regression model for psychological distress using the five-category physical activity classification; Table S4: Exploratory survey-weighted quasi-Poisson regression model for poor mental health days using the five-category physical activity classification.

## References

[1] Shuffler J., Lee K., Fields N., Graaf G., Cassidy J. (2023). Challenges Experienced by Rural Informal Caregivers of Older Adults in the United States: A Scoping Review. J. Evid.-Based Soc. Work.

[2] Wolff J.L., Spillman B.C., Freedman V.A., Kasper J.D. (2016). A National Profile of Family and Unpaid Caregivers Who Assist Older Adults With Health Care Activities. JAMA Intern. Med..

[3] Spiers G.F., Liddle J., Kunonga T.P., Whitehead I.O., Beyer F., Stow D., Welsh C., Ramsay S.E., Craig D., Hanratty B. (2021). What Are the Consequences of Caring for Older People and What Interventions Are Effective for Supporting Unpaid Carers? A Rapid Review of Systematic Reviews. BMJ Open.

[4] Hansen N.H., Bjerrekær L., Pallesen K.J., Juul L., Fjorback L.O. (2022). The Effect of Mental Health Interventions on Psychological Distress for Informal Caregivers of People with Mental Illness: A Systematic Review and Meta-Analysis. Front. Psychiatry.

[5] Sun Y., Iwagami M., Watanabe T., Sakata N., Sugiyama T., Miyawaki A., Tamiya N. (2021). Factors Associated with Psychological Distress in Family Caregivers: Findings from Nationwide Data in Japen. Geriatr. Gerontol. Int..

[6] Cui P., Ai J., Chen X., Cheng C., Shi J., Li S., Yang M., Chen C., Hu H. (2025). Dyadic Effects of Perceived Burden and Psychological Distress on Quality of Life among Chinese Advanced Cancer Patients and Their Caregivers. Sci. Rep..

[7] Musich S., Wang S.S., Kraemer S., Yeh C.S. (2023). The Association of Psychological Protective Factors with Caregiver Mental Health Outcomes. Geriatr. Nur..

[8] Mahindru A., Patil P., Agrawal V. (2023). Role of Physical Activity on Mental Health and Well-Being: A Review. Cureus.

[9] Myśliwiec N., Ciesielska A., Wojtczak M., Sieradzka A., Kot A., Różycki A., Pniak M., Mawlichanów M., Miklis P., Szerej K. (2025). The Impact of Physical Activity on Mental Health. Qual. Sport.

[10] Pickett A., Krukowski R., Werner N. (2023). Physical Activity Barriers and Intentions Of Alzheimer’s Disease (Ad) and Related Dementias (Ad/Adrd) Caregivers. Innov. Aging.

[11] Stowell E., Zhang Y., Castaneda-Sceppa C., Lachman M., Parker A.G. (2019). Caring for Alzheimer’s Disease Caregivers: A Qualitative Study Investigating Opportunities for Exergame Innovation. Proc. ACM Hum.-Comput. Interact..

[12] Denche-Zamorano Á., Barrios-Fernandez S., Gómez-Galán R., Franco-García J.M., Carlos-Vivas J., Mendoza-Muñoz M., Rojo-Ramos J., Vega-Muñoz A., Contreras-Barraza N., Gianikellis K. (2022). Associations between Physical Activity Level and Mental Health in the Spanish Population: A Cross-Sectional Study. Healthcare.

[13] Denche-Zamorano Á., Franco-García J.M., Carlos-Vivas J., Mendoza-Muñoz M., Pereira-Payo D., Pastor-Cisneros R., Merellano-Navarro E., Adsuar J.C. (2022). Increased Risks of Mental Disorders: Youth with Inactive Physical Activity. Healthcare.

[14] Pereira-Payo D., Denche-Zamorano Á., Mayordomo-Pinilla N., Franco-García J.M., Castillo-Paredes A., Garcia-Gordillo M.A., Rojo-Ramos J., Barrios-Fernández S. (2023). Higher Physical Activity Level and Perceived Social Support Is Associated with Less Psychological Distress in People with Anxiety. PeerJ.

[15] Denche-Zamorano Á., Pastor-Cisneros R., Carlos-Vivas J., Franco-García J.M., Pereira-Payo D., Barrios-Fernandez S., Rojo-Ramos J., Mendoza-Muñoz M. (2022). Associations between Psychological Distress, Perceived Social Support and Physical Activity Level in Spanish Adults with Depression. Healthcare.

[16] Denche-Zamorano Á., Mendoza-Muñoz M., Carlos-Vivas J., Muñoz-Bermejo L., Rojo-Ramos J., Pastor-Cisneros R., Giakoni-Ramírez F., Godoy-Cumillaf A., Barrios-Fernandez S. (2022). A Cross-Sectional Study on Self-Perceived Health and Physical Activity Level in the Spanish Population. Int. J. Environ. Res. Public Health.

[17] Denche-Zamorano A., Rodriguez-Redondo Y., Barrios-Fernandez S., Mendoza-Muñoz M., Rojo-Ramos J., Garcia-Gordillo M.A., Adsuar J.C., Muñoz-Bermejo L. (2023). Depression, Anxiety and Antidepressants and Anxiolytics Use in Spanish Informal Caregivers According to the Physical Activity Frequency: EHSS 2014–2020. Healthcare.

[18] Wolff J.L., Cornman J.C., Freedman V.A. (2025). The Number Of Family Caregivers Helping Older US Adults Increased From 18 Million To 24 Million, 2011–22. Health Aff..

[19] Cree R.A. (2020). Frequent Mental Distress among Adults, by Disability Status, Disability Type, and Selected Characteristics—United States, 2018. MMWR Morb. Mortal. Wkly. Rep..

[20] World Health Organization (2020). WHO Guidelines on Physical Activity and Sedentary Behaviour.

[21] Valero-Cantero I., Casals C., Corral-Pérez J., Barón-López F.J., Wärnberg J., Vázquez-Sánchez M.Á. (2022). Accelerometer-Measured Physical Activity, Inactivity, and Related Factors in Family Caregivers of Patients with Terminal Cancer. Int. J. Environ. Res. Public Health.

[22] Ahn S., Cobb S.J., Butler R., Abdullah S.M., Boltz M.P., Chung M.L., Roberson P.N.E., Lee J.-A., Zhao X., Anderson J.G. (2025). Effects of Physical Activity on Health Among Informal Caregivers of People With Cognitive Decline: A Systematic Review and Meta-Analysis. West. J. Nurs. Res..

[23] Barreda Gutiérrez M., Lera Torre J., Cantarero Prieto D. (2025). The Price of Care: Socioeconomic Determinants of Mental Health among Informal Caregivers in Europe. Eur. J. Public Health.

[24] Mehio-Sibai A., Feinleib M., Sibai T.A., Armenian H.K. (2005). A Positive or a Negative Confounding Variable? A Simple Teaching Aid for Clinicians and Students. Ann. Epidemiol..

[25] ter Riet G., Schuster N.A., Twisk J.W., Heymans M.W., Rijnhart J.J. (2021). Noncollapsibility and Its Role in Quantifying Confounding Bias in Logistic Regression. Hogesch. Van Amst..

[26] Zacharis T., Lyrakos G., Zisi V.Z. (2020). Physical Activity and Mental Health in Caregivers of Mental Ill Patients in Greece. Proceedings of the Journal of Human Sport and Exercise—2020—Spring Conferences of Sports Science.

[27] Koch L.C., Lunsky Y., John L.S. (2025). Physical Activity, Sedentary Behaviour, Sleep and Mental Wellbeing in Family Caregivers of Adults With Intellectual and/or Developmental Disabilities. J. Appl. Res. Intellect. Disabil..

[28] Pearce M., Garcia L., Abbas A., Strain T., Schuch F.B., Golubic R., Kelly P., Khan S., Utukuri M., Laird Y. (2022). Association Between Physical Activity and Risk of Depression: A Systematic Review and Meta-Analysis. JAMA Psychiatry.

[29] Guo Z., Li R., Lu S. (2022). Leisure-Time Physical Activity and Risk of Depression: A Dose-Response Meta-Analysis of Prospective Cohort Studies. Medicine.

[30] A’Azman S., Sung P., Malhotra R. (2024). Engagement in Physical Activity and Quality of Life Among Informal Caregivers of Older Adults. J. Aging Health.

[31] Ghim S., Ku B. (2022). The Prevalence of Health Problems and Their Association with Physical Activity in Caregivers of Children with Disabilities: 2018 National Health Interview Survey. Child Care Health Dev..

[32] Cho J., Sands L.P., Stevens A.B., Allore H.G., Horstman M.J. (2024). Profile of Caregiving Activities and Association With Physical Health Among Dementia Spousal Caregivers. Innov. Aging.

[33] Cardoso C., Lumini M.J., Martins T. (2025). Effects of Physical Exercise in Reducing Caregivers Burden: A Systematic Review. Front. Public Health.

[34] Marshall E., LaCaille R.A., LaCaille L.J., Lee J.E., Peterson E. (2022). Effects of Physical Activity Interventions for Caregivers of Adults: A Meta-Analysis. Health Psychol..

[35] Marquez D.X., Bustamante E.E., Kozey-Keadle S., Kraemer J., Carrion I. (2012). Physical Activity and Psychosocial and Mental Health of Older Caregivers and Non-Caregivers. Geriatr. Nur..

[36] Lindsay R.K., Vseteckova J., Horne J., Smith L., Trott M., De Lappe J., Soysal P., Pizzol D., Kentzer N. (2022). The Prevalence of Physical Activity among Informal Carers: A Systematic Review of International Literature. Sport Sci. Health.

[37] Ogunjesa B.A., De Andrade Leão O.A., Aguiñaga S., Schwingel A., Raj M. (2025). Associations of Physical Activity and Sedentary Behavior With Mental Distress According to Caregiver Status: Analysis of the Health Information National Trends Survey, 2022. Am. J. Health Promot..

[38] Sabo K., Chin E. (2021). Self-Care Needs and Practices for the Older Adult Caregiver: An Integrative Review. Geriatr. Nur..

[39] Malki S.T., Johansson P., Andersson G., Andréasson F., Mourad G. (2025). Caregiver Burden, Psychological Well-Being, and Support Needs among Swedish Informal Caregivers. BMC Public Health.

[40] Chong E., Crowe L., Mentor K., Pandanaboyana S., Sharp L. (2023). Systematic Review of Caregiver Burden, Unmet Needs and Quality-of-Life among Informal Caregivers of Patients with Pancreatic Cancer. Support. Care Cancer.

[41] Boyd K., Winslow V., Borson S., Lindau S.T., Makelarski J.A. (2022). Caregiving in a Pandemic: Health-Related Socioeconomic Vulnerabilities Among Women Caregivers Early in the COVID-19 Pandemic. Ann. Fam. Med..

[42] Savela R., Schwab U., Välimäki T. (2022). An Integrative Review of the Social Determinants of Mental Health among Older Caregivers. Nurs. Open.

