# Supplementary material for: Association of Physical Activity with Psychological Distress and Poor Mental Health Days Among Informal Caregivers of Older Adults: Findings from the 2023 BRFSS Caregiver Module
Source: Healthcare (Basel). 2026 Aug 27;14(17):2734. doi: 10.3390/healthcare14172734 (PMC13564686; doi:10.3390/healthcare14172734)
Supplement: Supplementary file 1 [file healthcare-14-02734-s001.zip › healthcare-4506960-supplementary.pdf]

Table S1. Missingness pattern among otherwise eligible caregivers (n = 2795)

Variable	Missing (n)	% of missing cases
Sex	0	0
Age (years)	0	0
Race/Ethnicity	51	1.8
Education	4	0.1
Household income	421	15.1
Marital status	15	0.5
Smoking status	20	0.7
Heavy alcohol consumption	35	1.3
Physical activity group (_TOTINDA)	6	0.2
Physical activity group (_PAREC3)	379	13.6
Poor mental health days	44	1.6
Body mass index	138	4.9
Weekly hours of caregiving	222	7.9
Duration of caregiving	81	2.9
Provided personal care	12	0.4
Provided household care	16	0.6
Relationship to the care recipient	20	0.7

Missingness was assessed among otherwise eligible caregivers, defined as respondents who reported being informal caregivers and whose care recipient's main condition was old age/infirmity/frailty or Alzheimer's disease, dementia, or another cognitive impairment disorder. Percentages were calculated using the otherwise eligible caregiver sample as the denominator (n = 2795). Because some respondents had missing information in more than one analytical variable, variable-specific missing counts do not sum to the total number of respondents excluded from the complete-case analysis.

Table S2. Comparison of included complete-case participants and otherwise eligible caregivers excluded due to missing data.

Variable		Included		Excluded		p
		n	%	n	%	
Sex	Male	773	43.3%	386	38.2%	0.009*
	Female	1012	56.7%	624	61.8%	
Race/Ethnicity	White non-Hispanic	1276	71.5%	688	71.7%	0.309
	Black non-Hispanic	81	4.5%	59	6.2%	
	American Indian / Alaska Native	30	1.7%	16	1.7%	
	Asian	120	6.7%	53	5.5%	
	Native Hawaiian / Pacific Islander	27	1.5%	12	1.3%	
	Other race	14	0.8%	3	0.3%	
	Multiracial	91	5.1%	42	4.4%	
	Hispanic	146	8.2%	86	9.0%	
Education	Elementary education	15	0.8%	11	1.1%	0.017*
	Some high school	60	3.4%	40	4.0%	
	High school graduate	364	20.4%	244	24.3%	
	Some college / technical school	545	30.5%	323	32.1%	
	College graduate	801	44.9%	388	38.6%	
Household income	< $10,000	46	2.6%	15	2.5%	0.543
	$10,000–<$15,000	36	2.0%	16	2.7%	
	$15,000–<$20,000	69	3.9%	24	4.1%	
	$20,000–<$25,000	88a	4.9%	35	5.9%	
	$25,000–<$35,000	184b	10.3%	72	12.2%	
	$35,000–<$50,000	275b	15.4%	101	17.1%	
	$50,000–<$75,000	310b	17.4%	88	14.9%	
	$75,000–<$100,000	267b	15.0%	76	12.9%	
	$100,000–<$150,000	270b	15.1%	95	16.1%	
	$150,000–<$200,000	123b	6.9%	34	5.8%	
	$200,000 or more	117b	6.6%	33	5.6%	
Marital status	Married	1093	61.2%	606	60.9%	0.138
	Divorced	270	15.1%	119	12.0%	
	Widowed	129	7.2%	86	8.6%	
	Separated	29	1.6%	19	1.9%	
	Never married	214	12.0%	138	13.9%	
	Member of unmarried couple	50	2.8%	27	2.7%	
Smoking status	Smoker	755	42.3%	419	42.3%	0.989
	Non-smoker	571	57.7%	1030	57.7%	
Heavy alcohol consumption	No	1665	93.3%	926	95.0%	0.075
	Yes	120	6.7%	49	5.0%	
Physical activity group	Met both recommendations	568	31.8%	198	31.4%	0.054
	Met aerobic recommendations	215	37.0%	660	34.1%	
	Met strength recommendations	69	3.9%	16	2.5%	
	Insufficient physical activity	82	4.0%	25	4.6%	
	Inactive	406	22.7%	177	28.1%	
Psychological distress	No	1514	84.8%	802	83.0%	0.218
	Yes	271	15.2%	164	17.0%	
Weekly hours of caregiving	Up to 8 hours per week	943	52.8%	400	50.8%	0.436
	9 to 19 hours per week	274	15.4%	135	17.1%	

	20 to 39 hours per week	220	12.3%	88	11.2%	
	40 hours or more	348	19.5%	165	20.9%	
Duration of caregiving	Less than 30 days	183	10.3%	104	11.2%	0.152
	1 month to less than 6 months	172	9.6%	90	9.7%	
	6 months to less than 2 years	418	23.4%	184	19.8%	
	2 years to less than 5 years	558	31.3%	284	30.6%	
	5 or more years	454	25.4%	267	28.7%	
Provided personal care	No	834	46.7%	528	52.9%	0.002*
	Yes	951	53.3%	470	47.1%	
Provided household care	No	334	18.7%	241	24.2%	0.001*
	Yes	1451	81.3%	753	75.8%	
Relationship to care recipient	Parent	821	46.0%	460	46.5%	0.606
	Spouse / Partner	272	15.2%	167	16.9%	
	Other relative	409	22.9%	214	21.6%	
	Non-relative or Family friend	283	15.9%	149	15.1%	
**Variable**		**Mean**	**SD**	**Mean**	**SD**	**p´**
Age (years)		59.92	14.68	60.49	15.48	0.097
Body mass index		28.62	6.58	28.19	6.13	0.239
Poor mental Health days		4.95	8.80	5.15	9.08	0.332

n: number of cases, SD: standard deviation, p: p-value of the Chi-squared test, p´: p-value of the Mann Whitney U test, * = p-value < 0.05. Values are presented as unweighted counts and percentages calculated among respondents with available data for each variable. Variable-specific denominators may differ because some excluded respondents had missing information for the characteristic being compared. P-values compare included and excluded otherwise eligible caregivers and were obtained using chi-square tests for categorical variables and Mann–Whitney U tests for continuous variables.

Table S3. Exploratory survey-weighted binary logistic regression model for psychological distress using the five-category physical activity classification.

Variable	Category	Odds Ratio	(95% CI)	p-value
**Physical activity group** (ref: *Met both*)	Met Aerobic	1.57	0.79–3.11	0.194
	Met Strength	1.81	0.58–5.60	0.305
	Insufficient PA	0.87	0.20–3.66	0.844
	Inactive	3.12	1.59–6.10	<0.001*
**Sex** (*ref: Male*)	Female	1.93	1.10–3.37	0.021*
**Race / ethnicity** (ref: *White non-Hispanic*)	Black non-Hispanic	0.43	0.15–1.26	0.125
	American Indian / Alaska Native	0.24	0.04–1.40	0.112
	Asian	0.70	0.15–3.23	0.647
	Native Hawaiian / Pacific Islander	0.78	0.08–7.56	0.829
	Other race	0.06	0.00–1.08	0.057
	Multiracial	1.25	0.41–3.83	0.697
	Hispanic	0.21	0.09–0.50	<0.001*
**Education** (ref: *Elementary education*)	Some high school	0.24	0.05–1.25	0.090
	High school graduate	0.30	0.06–1.44	0.135
	Some college / technical school	0.44	0.09–2.11	0.304
	College graduate	0.23	0.04–1.14	0.071
**Household income** (ref: *$50,000–<$75,000*)	< $10,000	0.50	0.11–2.28	0.373
	$10,000–<$15,000	0.54	0.14–2.13	0.378
	$15,000–<$20,000	2.66	0.81–8.75	0.108
	$20,000–<$25,000	4.30	1.21–15.30	0.024
	$25,000–<$35,000	4.08	1.51–11.02	0.006
	$35,000–<$50,000	1.02	0.35–2.96	0.973
	$75,000–<$100,000	0.50	0.15–1.71	0.269
	$100,000–<$150,000	1.08	0.39–2.99	0.884
	$150,000–<$200,000	2.56	0.83–7.90	0.103
	$200,000 or more	0.85	0.17–4.34	0.844
**Marital status** (ref: *Married*)	Divorced	1.89	0.88–4.09	0.105
	Widowed	0.86	0.31–2.34	0.763
	Separated	1.73	0.30–9.91	0.540
	Never married	1.37	0.55–3.45	0.501
	Member of unmarried couple	0.67	0.20–2.29	0.524
**Smoking** (ref: *Non-smoker*)	Smoker	2.83	1.64–4.88	<0.001*
**Heavy alcohol consumption** (ref: *No*)	Yes	3.38	1.26–9.11	0.016*
**Weekly hours of caregiving** (ref: *Up to 8 hours per week*)	9 to 19 hours per week	2.24	1.18–4.26	0.014*
	20 to 39 hours per week	1.26	0.48–3.29	0.636
	40 hours or more	0.93	0.45–1.95	0.858
**Duration of caregiving** (ref: *Less than 30 days*)	1 month to less than 6 months	0.49	0.18–1.36	0.173
	6 months to less than 2 years	0.47	0.21–1.09	0.079
	2 years to less than 5 years	0.28	0.12–0.68	0.005*
	5 or more years	1.23	0.53–2.87	0.630
**Relationship to care recipient** (ref: *Parent*)	Spouse / partner	1.61	0.64–4.05	0.310
	Other relative	0.74	0.39–1.40	0.354
	Non-relative / family friend	0.43	0.19–0.94	0.035*
**Provided personal care** (ref: *No*)	Yes	1.34	0.72–2.49	0.349

**Provided household care** (ref: *No*)	Yes	0.55	0.25–1.21	0.137
**Age (years)**		0.96	0.94–0.98	<0.001*
**Body mass index**		1.03	1.00–1.06	0.067

Table S4. Exploratory survey-weighted quasi-Poisson regression model for poor mental health days using the five-category physical activity classification.

Variable	Category	Rate Ratio	(95% CI)	p-value
**Physical activity group** (ref: *Met both*)	Met Aerobic	1.31	0.93–1.83	0.119
	Met Strength	1.20	0.74–1.96	0.455
	Insufficient PA	0.92	0.50–1.72	0.803
	Inactive	1.52	1.09–2.11	0.013*
**Sex** (ref: *Male)*	Female	1.37	1.07–1.77	0.014*
**Race/Ethnicity** (ref: *White non-Hispanic*)	Black non-Hispanic	0.62	0.38–1.02	0.061
	American Indian/Alaska Native	0.43	0.17–1.08	0.073
	Asian	0.74	0.36–1.52	0.413
	Native Hawaiian/Pacific Islander	0.64	0.19–2.17	0.479
	Other race	0.12	0.01–1.25	0.076
	Multiracial	1.05	0.60–1.81	0.873
	Hispanic	0.44	0.29–0.69	<0.001*
**Education** (ref: *Elementary education*)	Some high school	0.35	0.15–0.81	0.015*
	High school graduate	0.53	0.24–1.18	0.123
	Some college	0.67	0.31–1.46	0.312
	College graduate	0.41	0.18–0.91	0.028*
**Income** (ref: *$50,000–<$75,000*)	<$10,000	0.91	0.43–1.94	0.812
	$10,000–<$15,000	1.07	0.64–1.79	0.796
	$15,000–<$20,000	1.81	1.15–2.86	0.011*
	$20,000–<$25,000	1.88	1.10–3.23	0.021*
	$25,000–<$35,000	2.36	1.55–3.60	<0.001*
	$35,000–<$50,000	1.33	0.82–2.15	0.251
	$75,000–<$100,000	0.83	0.48–1.43	0.499
	$100,000–<$150,000	1.08	0.69–1.68	0.733
	$150,000–<$200,000	1.81	1.13–2.90	0.014*
	$200,000 or more	1.00	0.42–2.35	0.998
**Marital status** (ref: *Married*)	Divorced	1.39	0.97–2.00	0.075
	Widowed	1.05	0.61–1.80	0.864
	Separated	1.32	0.68–2.54	0.410
	Never married	1.26	0.83–1.91	0.274
	Unmarried couple	0.70	0.36–1.37	0.300
**Smoking** (ref: *Non-smoker*)	Smoker	1.70	1.30–2.21	<0.001*
**Heavy alcohol consumption** (ref: *No*)	Yes	1.54	1.00–2.38	0.050
**Weekly hours of caregiving** (ref: *Up to 8 hours per week*)	9 to 19 hours per week	1.36	1.01–1.83	0.045
	20 to 39 hours per week	1.12	0.72–1.73	0.617
	40 hours or more	0.89	0.62–1.28	0.530
**Duration of caregiving** (ref: *Less than 30 days*)	1 month to less than 6 months	0.70	0.43–1.15	0.162
	6 months to less than 2 years	0.73	0.49–1.09	0.122
	2 years to less than 5 years	0.57	0.38–0.86	0.007
	5 or more years	1.17	0.79–1.74	0.435
**Relationship to care recipient** (ref: *Parent*)	Spouse / partner	1.21	0.77–1.88	0.405
	Other relative	0.86	0.64–1.14	0.292
	Non-relative / family friend	0.60	0.40–0.90	0.014*
**Provided personal care** (ref: *No*)	Yes	1.21	0.90–1.61	0.203
**Provided household care**	Yes	0.78	0.55–1.12	0.185

(ref: *No*)			
**Age (years)**	0.97	0.96–0.98	<0.001*
**Body mass index**	1.01	1.00–1.02	0.086
